# Differentiation of *Mitragyna speciosa*, a narcotic plant, from allied *Mitragyna* species using DNA barcoding-high-resolution melting (Bar-HRM) analysis

**DOI:** 10.1038/s41598-021-86228-9

**Published:** 2021-03-24

**Authors:** Chayapol Tungphatthong, Santhosh Kumar J. Urumarudappa, Supita Awachai, Thongchai Sooksawate, Suchada Sukrong

**Affiliations:** 1 Research Unit of DNA Barcoding of Thai Medicinal Plants, Department of Pharmacognosy and Pharmaceutical Botany, Faculty of Pharmaceutical Sciences, Chulalongkorn University, Bangkok, 10330 Thailand; 2 Department of Pharmacology and Physiology, Faculty of Pharmaceutical Sciences, Chulalongkorn University, Bangkok, 10330 Thailand

**Keywords:** Biotechnology, Plant biotechnology, Plant molecular biology

## Abstract

*Mitragyna speciosa* (Korth.) Havil. [MS], or “kratom” in Thai, is the only narcotic species among the four species of *Mitragyna* in Thailand, which also include *Mitragyna diversifolia* (Wall. ex G. Don) Havil. [MD], *Mitragyna hirsuta* Havil. [MH], and *Mitragyna rotundifolia* (Roxb.) O. Kuntze [MR]. *M. speciosa* is a tropical tree belonging to the Rubiaceae family and has been prohibited by law in Thailand. However, it has been extensively covered in national and international news, as its abuse has become more popular. *M. speciosa* is a narcotic plant and has been used as an opium substitute and traditionally used for the treatment of chronic pain and various illnesses**.** Due to morphological disparities in the genus, the identification of plants in various forms, including fresh leaves, dried leaf powder, and finished products, is difficult. In this study, DNA barcoding combined with high-resolution melting (Bar-HRM) analysis was performed to differentiate *M. speciosa* from allied *Mitragyna* and to assess the capability of Bar-HRM assays to identify *M. speciosa* in suspected kratom or *M. speciosa*-containing samples. Bar-HRM analysis of PCR amplicons was based on the ITS2, *rbc*L, *trn*H*-psb*A*,* and *mat*K DNA barcode regions. The melting profiles of ITS2 amplicons were clearly distinct, which enabled the authentication and differentiation of *Mitragyna* species from allied species. This study reveals that DNA barcoding coupled with HRM is an efficient tool with which to identify *M. speciosa* and *M. speciosa*-containing samples and ensure the safety and quality of traditional Thai herbal medicines.

## Introduction

In Thailand, *Mitragyna speciosa* (Korth.) Havil*.* [MS]*, Mitragyna diversifolia* (Wall. ex G. Don) Havil. [MD], *Mitragyna hirsuta* Havil. [MH], and *Mitragyna rotundifolia* (Roxb.) O. Kuntze [MR], species belonging to the Rubiaceae family with specific medicinal importance, are commonly distributed in the central to southern regions^1–3^. Among these *Mitragyna* species, *M. speciosa* is a narcotic plant, and recently, it has been used as an opium substitute and for the treatment of addiction to morphine^4^. *Mitragyna speciosa,* “kratom” in Thai, is an evergreen tropical medicinal tree^5–8^ that is indigenous to Thailand, Malaysia and Indonesia. It has been used to treat various diseases, including malaria, fever, diarrhea and chronic pain^1,9,10^. *M. speciosa* shows gastrointestinal effects^10^, antidepressant activity^11^, and antioxidant and antibacterial properties^12^. Phytochemical studies have shown that its major chemical constituents are indole alkaloids, including mitragynine, 7-hydroxymitraginine, 5-desmethylmitragynine, 17-desmethyldihydro-mitragynine, speciogynine, speciocilliatine, and paynantheine^13,14^, along with other secondary metabolites, such as flavonoids, saponins, monoterpenes, triterpenoids, secoirioids and polyphenolic compounds^15^.

Kratom trees, which are native to Thailand, are used to sweeten the taste of traditional herbs. In 1943, kratom was regulated under the Kratom Act in Thailand, and later, it was revised and reclassified under the Narcotic Act of 1979^16^. The planting, growing, processing, export and import of kratom leaves are prohibited and now considered illegal^16^. Many countries have banned kratom or implemented severe, strict action or penalties for its possession. However, there are increasing reports of people mixing kratom leaves with pharmaceutical drugs, Coca-Cola cocktails, cough syrup and strange ingredients such as mosquito coil ash^17^. The demand for kratom is not often met due to the restriction and unavailability of the species in the required quantity in areas convenient for kratom processing. As a result, *M. speciosa* is substituted with other plant species, including *Mitragyna* species, or adulterated. The other species may have similar or different morphological characters and may differ in their chemical profiles. Although of natural origin and having been used for many years, traditional medicines are not yet recognized officially in many countries due to concerns about their safety, quality and efficacy^18,19^. The major reasons for the increase in such concerns are intentional or inadvertent substitution and adulteration^20–22^. The adulterant and substituted species may have different or lower pharmacological action compared with that of their authentic counterparts. Even species within the same genus may exhibit differences in pharmacological action. This inadvertent substitution and adulteration can cause intoxication and even death^20,22^. Thus, quality assurance in terms of the identity of herbal drugs used in traditional medicines is vital. The WHO normal rules for conventions and practices on research and assessment of traditional medicines state that the initial phase in assuring the safety and efficacy of traditional medicines is correct identification^23^.

Taxonomic identification of tree species can be challenging. Plants of the same species may vary in their morphology according to their growing environmental conditions, their age, and time, and closely related species may exhibit similar morphologies^24^. In the past decade, molecular identification tools have been broadly used for plant identification. DNA barcodes, short sections of DNA sequences, have been proven as an alternative tool for the identification of medicinal plants. In our previous studies, it was shown that sequences from the nuclear internal transcribed spacer (ITS) region can be used to differentiate *M. speciosa* from related species by the polymerase chain reaction-restriction fragment length polymorphism (PCR–RFLP) method^2^. However, the shortcomings of the PCR–RFLP approach are that it is time consuming and very limited in its ability to identify species when the samples are from incomplete specimens or damaged. More recently, several studies have shown that very closely related medicinal plant species can also be distinguished accurately by using DNA barcoding combined with high-resolution melting (Bar-HRM) analysis^21,25–27^.

Therefore, in this study, we aimed to develop Bar-HRM analysis for the differentiation of *M. speciosa* from related species and the investigation of *M. speciosa* in suspected kratom samples. The generated Bar-HRM analysis profiles enable verification of the authenticity of narcotic plant species for law enforcement.

## Results

### DNA analysis and primer design

DNA was successfully extracted from all collected *Mitragyna* species and suspected kratom samples. The genomic DNA concentrations of the *Mitragyna* species and suspected kratom samples were quantified using NanoDrop measurements (Table S1). The suspected kratom DNA samples were inconsistent in quality and quantity. The total DNA concentrations of all the *Mitragyna* species and suspected kratom samples were provided in Table S1. *rbc*L*, trn*H*-psb*A intergenic spacer*, mat*K*,* and ITS2 barcode sequences (Table 1) were amplified in all *Mitragyna* species and the suspected kratom samples. Positive PCR products with the expected length were confirmed with agarose gel electrophoresis. The *trn*H-*psb*A intergenic spacer was observed to have higher nucleotide variation (5.395%) than other regions, followed by ITS, *mat*K, and *rbc*L (3.780%, 0.658% and 0.069%, respectively) (Table 1).

**Table 1:** Evaluation of the four DNA barcode regions of *Mitragyna* species used in this study.

DNA barcode region	ITS	*mat*K	*rbc*L	*trn*H-*psb*A intergenic spacer
Length (bp)	607–608	1512–1518	1437	269–278
No. variable positions (bp)	23	10	1	15
Nucleotide variation (%)	3.780	0.658	0.069	5.395
Interspecific divergence (mean ± SD)	1.73 ± 0.93	0.13 ± 0.14	0.03 ± 0.04	0.57 ± 0.62
Intraspecific divergence (mean ± SD)	0.00 ± 0.00	0.00 ± 0.00	0.00 ± 0.00	0.00 ± 0.00
Average GC content (%, SD)	63.85	32.81	43.86	23.52

bp-base pair.

SD-standard deviation.

Multiple sequences of *M. speciosa* were used to design HRM primers. The HRM primer pairs were designed for the flanking regions of each DNA barcode region, yielding HRM amplicons ranging from 71 to 110 bp (Table 2; Fig. S1). The *trn*H-*psb*A intergenic spacer was observed to have the most variable sites (9 bp), which consisted of nine nucleotide insertion-deletion (indel) positions without single-nucleotide polymorphisms (SNPs), followed by ITS2 (1 bp), *mat*K (0 bp) and *rbc*L (0 bp) (Table 2). Highest variable characters (%) showed in *psb*A-*trn*H (8.82) by ITS2 (3.75), *mat*K (1.39) and *rbc*L (1.39) (Table 2). All *Mitragyna* species multiple sequence alignments were provided in supplementary figure (Fig. S2). However, when amplicons differ in just one or few nucleotides, they may present similar melting curve profiles with small shifts in T_m_ (Table 3). The GC content of the four HRM amplicons was calculated for the prediction of melting curve profiles. ITS2 had the highest average GC content at 63.27%, followed by *rbc*L, *mat*K and the *trn*H-*psb*A intergenic spacer at 46.18%, 36.97%, and 35.35%, respectively (Table 2). Bar-HRM analysis of four DNA loci of *M. speciosa* was performed using specific HRM primer pairs corresponding to the ITS2, *mat*K, *rbc*L and *trn*H-*psb*A intergenic spacer barcode regions. All HRM primer pairs were designed with conserved sequences with a 100% match to the target sites, which facilitated primer annealing and elongation initiation of DNA polymerase. The *mat*K and *rbc*L HRM primer pairs provided consistent amplicon sizes of 71 and 72 bp, respectively. The ITS2 and *trn*H-*psb*A intergenic spacer primers yielded variable-length amplicons of 79–80 and 101–110 bp, respectively (Table 2).

**Table 2:** Characteristics of the Bar-HRM amplicons from the four *Mitragyna* species.

DNA barcode region	ITS2	*mat*K	*rbc*L	*trn*H-*psb*A intergenic spacer
Expected product size in bp	79–80	71	72	101–110
Variable nucleotides	2	1	1	0
Insertion-deletion characters	1	0	0	9
Variable characters (%)	3.75	1.39	1.39	8.82
Average GC content (%, SD):	63.27(1.67)	36.97(0.70)	46.18(0.69)	35.35(0.55)

bp-base pair.

SD-standard deviation.

**Table 3:** The melting temperature values (T_m_) of four barcode regions derived from *Mitragyna* species.

Plant name	Melting temperature (T_m_) (°C)			
	ITS2	*mat*K	*rbc*L	*trn*H-*psb*A intergenic spacer
*M. speciosa*	83.9 ± 0.06	75.0 ± 0.06	79.0 ± 0.06	74.5 ± 0.06
*M. diversifolia*	85.2 ± 0.06	74.6 ± 0.00	78.2 ± 0.06	74.8 ± 0.06
*M. hirsuta*	85.2 ± 0.06	74.6 ± 0.00	78.2 ± 0.06	74.8 ± 0.06
*M. rotundifolia*	85.2 ± 0.06	74.6 ± 0.00	78.2 ± 0.06	74.8 ± 0.00

SD-standard deviation.

### Evaluation of HRM primer pairs for* Mitragyna species*

**Figure 1:**
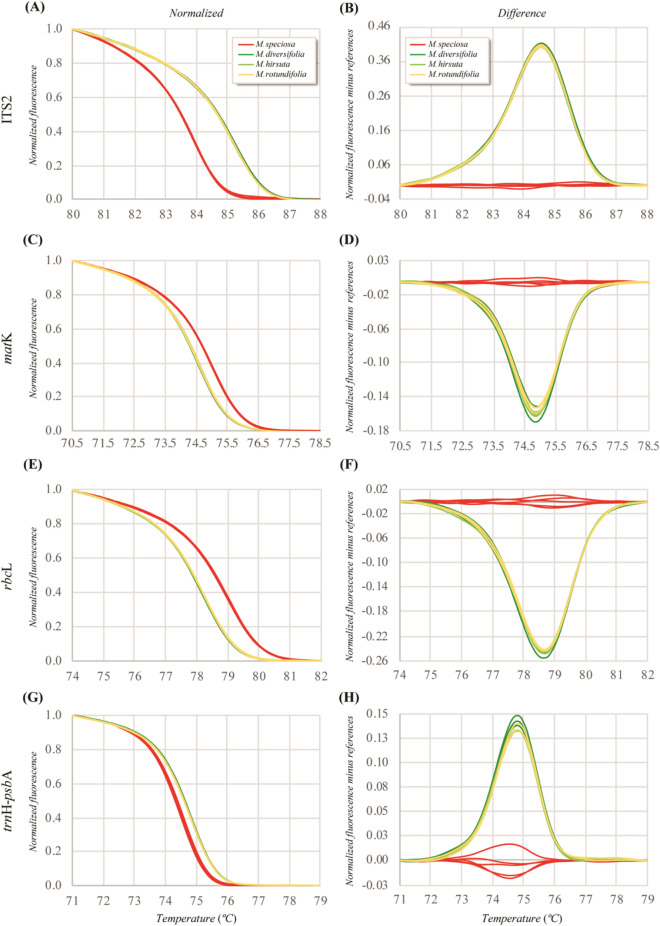
Bar-HRM analysis of *M. speciosa*, *M. diversifolia*, *M. hirsuta* and *M. rotundifolia*, shown in terms of normalized plots (**A**, **C**, **E**, **G**) and difference plots with *M. speciosa* as the reference (**B**, **D**, **F**, **H**).

The four HRM primer pairs Mit-ITS2, Mit-*mat*K, Mit-*rbc*L and Mit-*trn*H-*psb*A were designed and used for HRM analysis of the four *Mitragyna* species (Fig. S1). The Bar-HRM procedure was conducted to identify *Mitragyna* species. All samples were amplified with four different primer pairs prior to defining the T_m_ at the melting step in order to distinguish *M. speciosa* from related species (Table 3). The anticipated HRM amplicons from ITS2, *mat*K, *rbc*L and *trn*H-*psb*A were 79–80 bp, 71 bp, 72 bp and 101–110 bp, respectively (Table 2; Fig. S1). As shown in Fig. 1, the barcode regions of ITS2, *rbc*L, the *trn*H-*psb*A intergenic spacer and *mat*K in *M. speciosa* presented similar melting curve profiles and therefore could be visually differentiated from the normalized melting curves of other *Mitragyna* species. The melting curve analysis revealed different melting peaks, which could be used to discriminate *M. speciosa* from the other three species. To obtain the best visualization of very small differences between individual melting curves, Bar-HRM analysis of all four regions was conducted using Bio-Rad Precision Melting software to acquire HRM profiles and improve visualization for differentiation. Precision Melting software was used to calculate the difference in melting curves with *M. speciosa* as a reference. All the final normalized melting curves and separation of the different melting curves of the four *Mitragyna* species are shown in Fig. 1A-H. The HRM curve analysis showed slight melting temperature shifts between PCRs for all the *Mitragyna* species (Fig. 1; Table 3). However, the relative position and shape of the normalized melting curves were consistent compared to those of the differential melting curves (Fig. 1). Bar-HRM analysis of the four HRM primer pairs yielded a normalized plot and differential plot. The melting curves of the four *Mitragyna* species readily distinguished *M. speciosa* from the related species when using HRM analysis with four HRM primer pairs (Fig. 1). The melting profiles of the four *Mitragyna* plants could be separated into two clusters. All *M. speciosa* samples were collectively clustered (red line) (Fig. 1). In contrast, other allied species were clustered together (yellow, olive green and green lines) (Fig. 1). The differences in T_m_ between *M. speciosa* and related species for ITS2, *mat*K, *rbc*L and the *trn*H-*psb*A intergenic spacer were 1.3, 0.4, 0.8 and 0.3, respectively (Table 3). Although the four HRM primer pairs could be used to differentiate *M. speciosa* from related species, the ITS2 region, which provided the largest difference in T_m_, was selected for further investigation.

### Investigation of* M. speciosa* in suspected kratom samples

**Figure 2:**
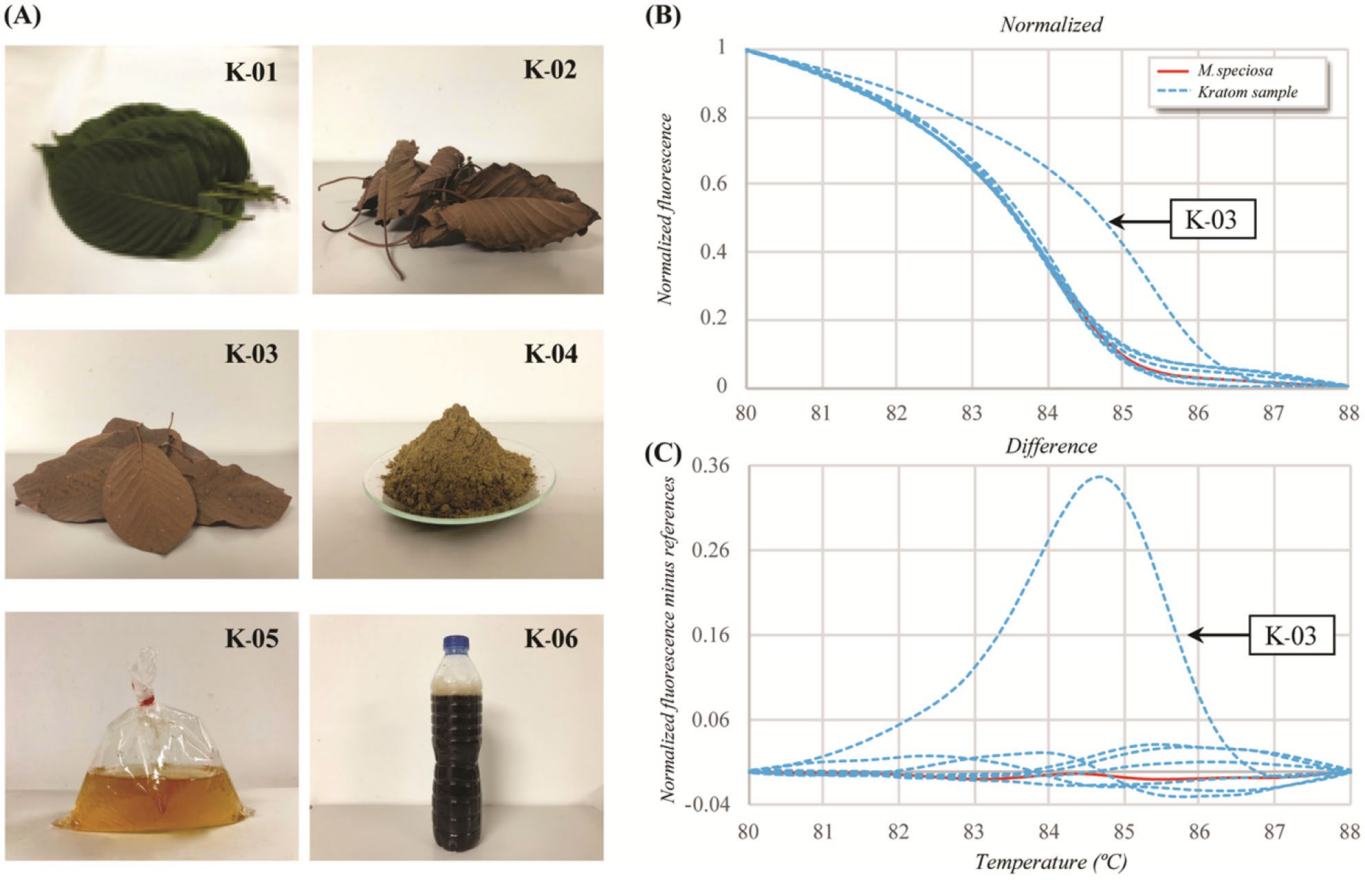
The investigation of suspected kratom samples via Bar-HRM analysis using the Mit_ITS2 primer pair. (**A**) Kratom samples in different forms. (**B**) and (**C**) Bar-HRM analysis of ITS2 regions of kratom samples, showing a normalized plot (**B**) and a difference plot (**C**).

According to the significant differences in the melting profiles of *M. speciosa* and other related *Mitragyna* species detecting using the Mit-ITS2 primer pair, the Bar-HRM approach was used to detect *M. speciosa* in suspected kratom samples (Table 4; Fig. 2A). In the Bar-HRM analysis of normalized (Fig. 2B) and differential curves using the *M. speciosa* curve as the reference (Fig. 2C), five out of six suspected kratom samples, namely, K-01, K-02, K-04, K-05 and K-06, had T_m_ values ranging from 83.9 to 84.0 °C, which were similar to those of the references (Table 4). On the other hand, kratom sample K-03 had the differential curve with the highest distinct T_m_ value of 85.2 °C, placing the sample in the non-*M. speciosa* cluster (Table 4; Fig. 2B,C). The melting curves of the six suspected kratom samples formed two clusters: the *M. speciosa* cluster (K-01, K-02, K-04, K-05 and K-06) and the non-*M. speciosa* cluster (K-03) (Fig. 2B,C). However, all HRM amplicons were sequenced and NCBI BLAST searched against the GenBank database for species confirmation. The NCBI BLAST results for suspected kratom samples K-01, K-02, K-04, K-05 and K-06 indicated a very close match to *M. speciosa*, with the highest query coverage and maximum identity, and sample K-03 matched to non-*M. speciosa* (Table 4; Fig. S3).

**Table 4:** Investigation of suspected kratom samples using HRM clustering and query matching of ITS2 sequences in the NCBI database.

Sample code	Type of kratom sample	Melting temperature (T_m_) (°C)	HRM cluster	BLAST identity
K-01	Fresh leaves	84.0	*M. speciosa*	*M. speciosa*
K-02	Dried leaves	83.9	*M. speciosa*	*M. speciosa*
K-03	Dried leaves	85.2	Non*-M. speciosa*	Non*-M. speciosa*
K-04	Powder	83.9	*M. speciosa*	*M. speciosa*
K-05	Juice	83.9	*M. speciosa*	*M. speciosa*
K-06	Cocktail	84.0	*M. speciosa*	*M. speciosa*

## Discussion

*M. speciosa* or kratom has a very long history of traditional usage in Southeast Asia. Because kratom is increasingly considered a less expensive and more readily available substitute drug^5^, it has reportedly gained more attention as an alternative to opium, at least in Thailand. Although kratom is a narcotic and banned in certain countries because of its opioid-like effects^5,6^, people living in rural areas continue to believe that kratom consumption is less harmful than the consumption of other banned drugs; in fact, its usage has not been reported to have any significant health threats, in contrast to opiate misuse^9,16,28,29^.

In the past decade, molecular analysis has become an acceptable tool for the authentication of medicinal plants. Many studies have revealed DNA barcode regions that can discriminate plants at the genus or species level^30^. In this study, four universal DNA barcode regions, *rbc*L, the *psb*A*-trn*H intergenic spacer*, mat*K, and ITS2, were evaluated for their applicability in identifying four *Mitragyna* species. The ITS2 region yielded the highest interspecific divergence among the four species of *Mitragyna* and revealed the highest nucleotide variation among them (Table 1). Indeed, the ITS2 barcode region is one of the most variable regions in angiosperms and can be used for species differentiation^30,31^. Recently, Bar-HRM analysis has been successful in the identification of various medicinal plants^32^. The advantage of HRM is its capacity to screen variations in specific regions that would not be identified by Sanger sequencing; moreover, using HRM analysis of melting temperatures can allow any different nucleotides in a sequence to be detected^33^. Recent studies have shown that Bar-HRM analysis can be effective in providing consistent shapes and profiles of melting curves that correspond to very closely related medicinal plant species and allow the authentication of herbal products^21,25–27^. Nucleotide composition and DNA length are affected by the dissociation step of double-stranded amplicons in HRM analysis. The characteristics of sequences influence the melting curve profiles and differentiation of melting temperature (T_m_) values in Bar-HRM analysis. The optimal sequence length for obtaining accurate results from Bar-HRM analysis is 300 bp or less^34^. In our study, the lengths of the HRM amplicons were in the range of 71 to 110 bp, which is consistent with the findings of previous reports. High nucleotide consensus at HRM primer sites is critical for the annealing of primers and elongation of amplicons by DNA polymerase^35^. Our HRM primers designed for the four DNA regions showed 100% consensus in the flanking regions of each variable DNA site in the four *Mitragyna* species, which promoted the efficiency of DNA amplification.

Further, we developed a Bar-HRM analysis method that can clearly differentiate *M. speciosa* from closely related species, including *M. diversifolia*, *M. hirsuta* and *M. rotundifolia,* and identified them directly in suspected kratom samples. In our study, the four newly designed HRM primer pairs (Fig. S1) were successfully used for the differentiation of *M. speciosa* from related species. Although all HRM analyses of the four regions displayed satisfactory discrimination of *M. speciosa* from allied *Mitragyna* species, the highest differential melting temperature (T_m_) was observed in the Bar-HRM analysis of ITS2. The ITS2 region was used as a target for HRM analysis and has been demonstrated to be an effective tool for the detection and quantification of several plants, such as plants in the genus *Sideritis*^36^*,* nine herbal teas^37^, plants in the genus *Artemisia*^38^, two *Ophiocordyceps* species^39^, and edible plants^27^. The results from the Mit-*rbc*L and Mit-*mat*K primer pairs showed a higher T_m_ for *rbc*L HRM amplicons than for *mat*K HRM amplicons. However, their amplicon sizes were very similar, and they showed the same type of nucleotide substitution (*mat*K: C → T and *rbc*L: G → T). This study showed that the ITS2 amplicons had the highest GC content (63.27%), the *trn*H-*psb*A intergenic spacer amplicons had the lowest GC content (35.35%) (Table 2), and nucleotide variations in these regions exhibited the largest difference in T_m_ among the four *Mitragyna* species (Table 3). In our analysis, GC content was the most important metric for choosing candidate DNA barcode regions for combination with HRM analysis. A higher GC content will lead to a larger T_m_ difference. Therefore, the GC content of nucleotide variations among species is the key factor for the authentication of plant species.

In the investigation of suspected kratom samples, five out of six samples (K-01, 02, 04, 05, and 06) clustered with *M. speciosa*, and only one suspected sample (K-03) was included in the non-*M. speciosa* cluster (Table 4; Fig. 2). Suspected kratom sample K-03 clustered with non-*M. speciosa,* consistent with the NCBI BLAST result (Table 4). This study proves the efficiency of Bar-HRM analysis for the identification of raw material and highly processed samples. The closed-tube system can be simultaneously performed without a post-PCR assay. Moreover, this method is suitable for routine analysis in the laboratory without the need for high expertise. Our research provides a valuable tool for the characterization of *Mitragyna* species that can be useful for quick one-step real-time PCR for the simultaneous identification of suspected kratom samples. Furthermore, this analysis is potentially useful for the identification and differentiation of closely related plant species in mixed-plant samples.

## Conclusion

To the best of our knowledge, this is the first study of DNA barcoding coupled with HRM analysis for the differentiation of *M. speciosa*, a narcotic species, from closely related species and for the investigation of suspected kratom samples. ITS2 best reflected the relationships between the four *Mitragyna* species that we tested and can be used consistently to determine species identity. The Bar-HRM results from the Mit-ITS2 primer pair indicated that ITS2 can be used as an effective DNA barcode marker for *Mitragyna* species. Bar-HRM analysis provides a simple, sensitive, and reliable method for the investigation of *M. speciosa* and suspected kratom samples for routine analysis in forensic laboratories. These outcomes will aid in the authentication of *M. speciosa* in suspected kratom samples as well as deliver a new method with which to identify and differentiate suspected kratom samples, ensure safety and quality control for traditional medicine and identify narcotic plant species for law enforcement.

## Materials and methods

### Plant materials and suspected kratom samples

We, under the permission license to Faculty of Pharmaceutical Sciences, Chulalongkorn University, received the approval and permission documents by the Thai Food and Drug Administration (FDA) (License No. 8/2563) for conducting the experiment and field studies on plants. The protocols for plant collection and field studies of kratom (*M. speciosa*) were conducted by following The Narcotic Act. BE. 2522 of Thailand. Multiple accessions of the four *Mitragyna* species, namely, *M. speciosa*, *M. diversifolia*, *M. hirsuta* and *M. rotundifolia,* were collected from natural sources in various parts of Thailand. Sample details along with their voucher numbers and place of collection are provided in Table S2. All samples were identified by an expert taxonomist, Assoc. Prof. Thatree Phadungcharoen, Rangsit University, Thailand. Each voucher specimen was assigned a specific number and deposited in the Museum of Natural Medicine, Chulalongkorn University, Thailand. These authenticated plant specimens were further applied to perform Bar-HRM analysis using the nuclear region of the ITS and three chloroplast regions, namely, *rbc*L, the *trn*H-*psb*A intergenic spacer and *mat*K. Five different forms of suspected kratom samples presented as *M. speciosa,* including fresh leaves (K-01), dried leaves (K-02 and K-03), powder (K-04), juice (K-05), and a cocktail (K-06), were collected from anonymous sources in the southern part of Thailand (Fig. 2A; Table S3).

### DNA isolation from authentic* Mitragyna species* and suspected kratom samples

For genomic DNA extraction, 100 mg of fresh leaves of authentic *Mitragyna* species was isolated using a DNeasy Plant Kit (Qiagen, Germany) according to the manufacturer’s instructions. Total genomic DNA extraction from suspected kratom samples was performed using a DNeasy Plant Pro Kit according to the manufacturer’s guidelines. DNA isolation from the kratom juice and kratom cocktail was modified by adding a centrifugation step at 16,000 rpm for 20 min prior to DNA extraction. Approximately 20 mg of sedimented pellet in the bottom of the tube was extracted with the abovementioned protocols. Extracted DNA was further purified using a GENECLEAN II Kit (MP Biomedicals, USA). The quality and quantity of genomic DNA were determined using agarose gel electrophoresis and a NanoDrop spectrophotometer (Thermo Fisher, USA). All DNA was kept at − 20 °C prior to more in-depth analysis.

### Sequence analysis and HRM primer design

All sequences from the plastid DNA, including *mat*K, *rbc*L and the *trn*H-*psb*A intergenic spacer, along with the ITS2 sequence of *Mitragyna* plants were retrieved from our previous study^2,40^ (Table S2). Multiple alignments of sequences were performed using MEGA7^41^. Interspecific divergence was calculated using MEGA7 with the Kimura 2-parameter (K2P) distance model and pairwise deletion algorithm. Variable characters and GC content were calculated for all DNA barcode regions in the *Mitragyna* species (Table 1). All four DNA barcode regions were adopted to design the HRM primer pairs based on the consensus flanking region, which covered enough variable sites to allow the differentiation of narcotic species from nonnarcotic species. All the primers used in this were mentioned in Table S4. The expected sequences of HRM amplicons, nucleotide variation and average GC percentage were calculated for further analysis (Table 2).

### Bar-HRM analysis

PCR and melting analysis were implemented on a CFX96 Real-Time PCR Detection System (Bio-Rad, USA). The PCRs were performed in a total volume of 20 μL, containing 1 × SsoFast EvaGreen Supermix (Bio-Rad, USA), 0.5 μM forward and reverse primers of each DNA barcode region, and 10 ng of genomic DNA from each plant species or suspected kratom sample. The thermal cycling conditions were predenaturation at 95 °C for 1 min, followed by 44 cycles of 95 °C for 15 s, 63 °C for 20 s and 72 °C for 20 s. The fluorescence signal was obtained by the fluorescence (FAM) channel at the end of each extension step. Subsequently, HRM analysis was performed by increasing the temperature by 0.1 °C increments from 60 °C to 90 °C. For HRM analysis, the HRM amplicons were melted in ramped steps from 65 °C to 90 °C in 0.1 °C increments. Fluorescence intensity was measured at every increasing step. The HRM curves were analyzed using CFX Manager software (version 3.1 upgrade) and Precision Melt Analysis software (version 1.3 upgrade)^42^. HRM analysis was performed in triplicate for each reaction.

### Testing of suspected kratom samples

Total genomic DNA was isolated from each suspected kratom sample and then subjected to HRM analysis with the Mit-ITS2 primer pair for the ITS2 region for identification via melting temperature (T_m_). HRM amplicons of each sample were sequenced on an ABI 3730XL DNA Analyzer. NCBI BLAST analysis was performed against the GenBank database to authenticate the plant species.

## Supplementary Information

Supplementary Information 1.

Supplementary Information 2.

Supplementary Information 3.

Supplementary Information 4.

Supplementary Information 5.

Supplementary Information 6.

Supplementary Information 7.
