# Supplementary material for: Differentiation of Mitragyna speciosa, a narcotic plant, from allied Mitragyna species using DNA barcoding-high-resolution melting (Bar-HRM) analysis
Source: Sci Rep. 2021 Mar 24;11:6738. doi: 10.1038/s41598-021-86228-9 (PMC7990970; doi:10.1038/s41598-021-86228-9)
Supplement: Supplementary file 2 — Supplementary Information 2. [file 41598_2021_86228_MOESM2_ESM.pdf]

Tittle	**Differentiation of *Mitragyna speciosa*, a narcotic plant, from allied *Mitragyna* species using DNA barcoding-high-resolution melting (Bar-HRM) analysis**
Authors	Chayapol Tungphatthong[1,3], Santhosh Kumar J. Urumarudappa1[,3], Supita Awachai[1], Thongchai Sooksawate[2] and Suchada Sukrong[1]*
Affiliation	[1]Research Unit of DNA Barcoding of Thai Medicinal Plants, Department of Pharmacognosy and Pharmaceutical Botany, Faculty of Pharmaceutical Sciences, Chulalongkorn University, Bangkok 10330, Thailand
	[2]Department of Pharmacology and Physiology, Faculty of Pharmaceutical Sciences, Chulalongkorn University, Bangkok 10330, Thailand
	[3]These authors contributed equally: Chayapol Tungphatthong and Santhosh Kumar J. Urumarudappa
*Corresponding author	Professor Suchada Sukrong, Ph.D.
	Research Unit of DNA Barcoding of Thai Medicinal Plants,
	Department of Pharmacognosy and Pharmaceutical Botany,
	Faculty of Pharmaceutical Sciences, Chulalongkorn University,
	Bangkok 10330, Thailand
	Phone: +6681-819-6742, Fax: +6622-558-227
	Email: suchada.su@chula.ac.th

Internal Transcribed Spacer (ITS)

```
M. speciosa      TCGAATCCTGCAAAACGCACGACCGTGAACCCGTGTTAACACCCGGGCGTCGGGTGGCCG  60
M. diversifolia  TCGAATCCTGCAAAACGCACGACCGTGAACCCGTGTTAACAGCCGGGCGTCGGGTGGCCG  60
M. hirsuta       TCGAATCCTGCAAAACGCACGACCGTGAACCCGTGTTAACAGCCGGGCGTCGGGTGGCCG  60
M. rotundifolia  TCGAATCCTGCAAAACGCACGACCGTGAACCTGTGTTAACAGCCGGGCGTCGGGTGGCCG  60
                 *******************************^*********^******************

M. speciosa      TGGAGACTAAGCCCTCCCTTCCTTCCCGGCGCTCCCCGCGCGCTCGTCGCGCGGAAAACG  120
M. diversifolia  TGGAGACTAAGCCCTCCCTTCCTTCCCGGCGCTCCCCGCGTGCTCGTCGCGCGG-AAACG  119
M. hirsuta       TGGAGACTAAGCCCTCCCTTCCTTCCCGGCGCTCCCCGCGTGCTCGTCGCGCGG-AAACG  119
M. rotundifolia  CGGAGAATAAGCCCTCCCTTCCTTCCCGGCGCTCCCCGCGTGCTCGTCGCGCGG-AAACG  119
                  *****^*********************************^*************^*****

M. speciosa      TAACTCAAACCCGGCGCGGAACGCGCCAAGGAAAACTCAATAGGACTGCCA-AACCCCCC  179
M. diversifolia  TAACTCAAACCCGGCGCGGAACGCGCCAAGGAAAACTCAATAGGACTGCCGAAACCCCCC  179
M. hirsuta       TAACTCAAACCCGGCGCGGAACGCGCCAAGGAAAACTCAATAGGACTGCCGAAACCCCCC  179
M. rotundifolia  TAACTCAAACCCGGCGCGGAACGCGCCAAGGAAAACTCAATAGGACTGCCATGCCCCCCC  179
                 **************************************************^^^^******

M. speciosa      GATGCCCCGTTCGCGGTGCGCTCGAGGGTGCTGCGGCGCCTGTCTAATCCAAACGACTCT  239
M. diversifolia  GATGCCCCGTTCGCGGTGCGCTCGAGGGTGCTGCGGCGCCTGTCTAATCCAAACGACTCT  239
M. hirsuta       GATGCCCCGTTCGCGGTGCGCTCGAGGGTGCTGCGGCGCCTGTCTAATCCAAACGACTCT  239
M. rotundifolia  GATGCCCCGTTCGCGGTGCGCTCGAGGGTGCTGCGGCGCCTGTCTAATCCAAACGACTCT  239
                 ************************************************************

M. speciosa      CGGCAACGGATATCTCGGCTCTCGCATCGATGAAGAACGTAGCGAAATGCGATACTTGGT  299
M. diversifolia  CGGCAACGGATATCTCGGCTCTCGCATCGATGAAGAACGTAGCGAAATGCGATACTTGGT  299
M. hirsuta       CGGCAACGGATATCTCGGCTCTCGCATCGATGAAGAACGTAGCGAAATGCGATACTTGGT  299
M. rotundifolia  CGGCAACGGATATCTCGGCTCTCGCATCGATGAAGAACGTAGCGAAATGCGATACTTGGT  299
                 ************************************************************

M. speciosa      GTGAATTGCAGAATCCCGTGAACCATCGAGTCTTTGAACGCAAGTTGCGCCCGAAGCCAT  359
M. diversifolia  GTGAATTGCAGAATCCCGTGAACCATCGAGTCTTTGAACGCAAGTTGCGCCCGAAGCCAT  359
M. hirsuta       GTGAATTGCAGAATCCCGTGAACCATCGAGTCTTTGAACGCAAGTTGCGCCCGAAGCCAT  359
M. rotundifolia  GTGAATTGCAGAATCCCGTGAACCATCGAGTCTTTGAACGCAAGTTGCGCCCGAAGCCAT  359
                 ************************************************************

M. speciosa      CAGGCCAAGGGCACGTCTGCCTGGGCGTCACGCATCGCGTCGCCGCCCCCACCTGTCGTG  419
M. diversifolia  CCGGCCAAGGGCACGTCTGCCTGGGCGTCACGCATCGCGTCGCCGCCCCCACCTGTCGTG  419
M. hirsuta       CCGGCCAAGGGCACGTCTGCCTGGGCGTCACGCATCGCGTCGCCGCCCCCACCTGTCGTG  419
M. rotundifolia  CAGGCCAAGGGCACGTCTGCCTGGGCGTCACGCATCGCGTCGCCGCCCCCACCTGTCGTG  419
```

```
                  *^**********************************************************

M. speciosa      TGGGGCGGCGGATATTGGCCTCCCGTGCCGTGAGGTGCGGCCGGCCTAAATGCGAGTCCT  479
M. diversifolia  TGGGGCGGCGGATATTGGCCTCCCGTGCCGTAAGGTGCGGCCGGCCTAAATGCGAGTCCT  479
M. hirsuta       TGGGGCGGCGGATATTGGCCTCCCGTGCCGTAAGGTGCGGCCGGCCTAAATGCGAGTCCT  479
M. rotundifolia  TGGGGCGGCGGATATTGGCCTCCCGTGCCGTAAGGTGCGGCCGGCCTAAATGCGAGTCCT  479
                 *******************************^****************************

M. speciosa      CGGCGAGGGACGTCACGACGAGTGGTGGTTGAATGACCCGACTCGATTTCTGTCGTGCCG  539
M. diversifolia  CGGCGAGGGACGTCACGACGAGTGGTGGT-GAACGCCCCGACTCGATTTCTGTCGTGCCG  538
M. hirsuta       CGGCGAGGGACGTCACGACGAGTGGTGGT-GAACGCCCCGACTCGATTTCTGTCGTGCCG  538
M. rotundifolia  CGGCGAGGGACGTCACGACGAGTGGTGGT-GAACGCCCCGACTCGTTTTCTGTCGTGCCG  538
                 *****************************^***^*^*********^**************

M. speciosa      GCTTCCCTCGCTGTCTCCGGCTCCACGGATGACCCTCGTGCGCGCTCTCTCCGGAGTCGC  599
M. diversifolia  GCTTCCCTCGCTGTCTCCGGCTCTGCGGATGACCCTCGTGCGCGCTCTCTCCGGAGTCGC  598
M. hirsuta       GCTTCCCTCGCTGTCTCCGGCTCTGCGGATGACCCTCGTGCGCGCTCTCTCCGGAGTCGC  598
M. rotundifolia  GCTTCCCTCGCTGTCTCCGGCTCTGCGGATGACCCTTGTGCGCGCACTCTCCGGAGCCGC  598
                 ***********************^^***********^********^**********^***

M. speciosa      GCCCCGACC  608
M. diversifolia  GCCCCGAAC  607
M. hirsuta       GCCCCGAAC  607
M. rotundifolia  GCCCCGACC  607
                 *******^*
```

***psb*A – *trn*H Intergenic Spacer**

```
M. speciosa      CCTCTACTCTAATTTTATATATATATTTTTTTTTTAATTTCATATTCTATTTATTTAATA  60
M. diversifolia  CCTCTACTCTAATTT--TTTATATTTTTTTTTTTGAATTTCATATTCTATTTATTTAATA  58
M. hirsuta       CCTCTACTCTAATTT--TTTATATTTTTTTTTTTGAATTTCATATTCTATTTATTTAATA  58
M. rotundifolia  CCTCTACTCTAATTT--TTTATATTTTTTTTTTTGAATTTCATATTCTATTTATTTAATA  58
                 ***************^^*^*****^*********^*************************

M. speciosa      TTTAAATTCAAAAATTTCGTTTCGAACATTTTCTTTGCTTTTCTTTAAAAAAAAACATCG  120
M. diversifolia  TTTAAATTCAAAAATTTCGTTTCGAACATTTTCTTTGCTTTTCTTT-AAAAAAAACATCG  117
M. hirsuta       TTTAAATTCAAAAATTTCGTTTCGAACATTTTCTTTGCTTTTCTTT-AAAAAAAACATCG  117
M. rotundifolia  TTTAAATTCAAAAATTTCGTTTCGAACATTTTCTTTGCTTTTCTTT-AAAAAAAACATCG  117
                 **********************************************^*************

M. speciosa      ATATTTAAGTCCGAGTACTAATAAAAATACTAGAATAAATAAAAAGAAAATAAAGGAGCAA  180
M. diversifolia  ATATTTAAGTCCGAGTACTAATAAAAATACTAGAATAAATAAAAAGAAAATAAAGGAGCAA  177
M. hirsuta       ATATTTAAGTCCGAGTACTAATAAAAATACTAGAATAAATAAAAAGAAAATAAAGGAGCAA  177
M. rotundifolia  ATATTTAAGTCCGAGTACTAATAAAAATACTAGAATAAATAAAAAGAAAATAAAGGAGCAA  177
                 ************************************************************

M. speciosa      TAAAGCCCTCTTATCTATCTTATATAGAAGAAGAAGGAAATTATTGCTCCTTTATTTTTC  240
M. diversifolia  TAAAGCCCTCTTATCTATCTTATATAGAAGAAGAAGGAAATTATTGCTCCTTTATTTTTC  237
M. hirsuta       TAAAGCCCTCTTATCTATCTTATATAGAAGAAGAAGGAAATTATTGCTCCTTTATTTTTC  237
M. rotundifolia  TAAAGCCCTCTTATCTATCTTATATAGAAGAAGAAGGAAATTATTGCTCCTTTATTTTTC  237
                 ************************************************************

M. speciosa      AATAACTCCTATACAATAAGAACAATAAGACCGGGGTCTTATCCATTTATAGATGGAGCA  300
M. diversifolia  AATAACTCCTATACAATAAGA---------CCGGGGTCTTATCCATTTATAGATGGAGCA  291
M. hirsuta       AATAACTCCTATACAATAAGA---------CCGGGGTCTTATCCATTTATAGATGGAGCA  291
M. rotundifolia  AATAACTCCTATACAATAAGA---------CCGGGGTCTTATCCATTTATAGATGGAGCA  291
                 *********************^^^^^^^^^******************************

M. speciosa      TCTATAGCAGCTAGGTCTA  319
M. diversifolia  TCTATAGCAGCTAGGTCTA  310
M. hirsuta       TCTATAGCAGCTAGGTCTA  310
M. rotundifolia  TCTATAGCAGCTAGGTCTA  310
                 *******************
```

***rbc*L**

```
M. speciosa      ATGTCACCACAAACAGAAACTAAAGCAAGTGTTGGATTCAAAGCTGGTGTTAAAGAGTAC  60
M. diversifolia  ATGTCACCACAAACAGAAACTAAAGCAAGTGTTGGATTCAAAGCTGGTGTTAAAGAGTAC  60
M. hirsuta       ATGTCACCACAAACAGAAACTAAAGCAAGTGTTGGATTCAAAGCTGGTGTTAAAGAGTAC  60
M. rotundifolia  ATGTCACCACAAACAGAAACTAAAGCAAGTGTTGGATTCAAAGCTGGTGTTAAAGAGTAC  60
                 ************************************************************

M. speciosa      AAATTGACTTATTATACTCCTGAATACGAAACCAAAGAGACTGATATCTTAGCAGCATTC  120
M. diversifolia  AAATTGACTTATTATACTCCTGAATACGAAACCAAAGAGACTGATATCTTAGCAGCATTC  120
M. hirsuta       AAATTGACTTATTATACTCCTGAATACGAAACCAAAGAGACTGATATCTTAGCAGCATTC  120
M. rotundifolia  AAATTGACTTATTATACTCCTGAATACGAAACCAAAGAGACTGATATCTTAGCAGCATTC  120
```

```
                ************************************************************

M. speciosa     CGAGTAACTCCTCAACCTGGAGTTCCACCTGAAGAAGCAGGGGCCGCGGTAGCTGCCGAA  180
M. diversifolia CGAGTAACTCCTCAACCTGGAGTTCCACCTGAAGAAGCAGGGGCCGCGGTAGCTGCCGAA  180
M. hirsuta      CGAGTAACTCCTCAACCTGGAGTTCCACCTGAAGAAGCAGGGGCCGCGGTAGCTGCCGAA  180
M. rotundifolia CGAGTAACTCCTCAACCTGGAGTTCCACCTGAAGAAGCAGGGGCCGCGGTAGCTGCCGAA  180
                ************************************************************

M. speciosa     TCTTCTACTGGTACATGGACAACTGTGTGGACCGATGGACTTACCAGCCTTGATCGTTAC  240
M. diversifolia TCTTCTACTGGTACATGGACAACTGTGTGGACCGATGGACTTACCAGCCTTGATCGTTAC  240
M. hirsuta      TCTTCTACTGGTACATGGACAACTGTGTGGACCGATGGACTTACCAGCCTTGATCGTTAC  240
M. rotundifolia TCTTCTACTGGTACATGGACAACTGTGTGGACCGATGGACTTACCAGCCTTGATCGTTAC  240
                ************************************************************

M. speciosa     AAAGGACGATGCTACCATATCGAGCCAGTTCCTGGAGAAGAAGATCAATATATTGCTTAT  300
M. diversifolia AAAGGACGATGCTACCATATCGAGCCAGTTCCTGGAGAAGAAGATCAATATATTGCTTAT  300
M. hirsuta      AAAGGACGATGCTACCATATCGAGCCAGTTCCTGGAGAAGAAGATCAATATATTGCTTAT  300
M. rotundifolia AAAGGACGATGCTACCATATCGAGCCAGTTCCTGGAGAAGAAGATCAATATATTGCTTAT  300
                ************************************************************

M. speciosa     GTAGCTTACCCTTTAGACCTTTTTGAAGAAGGTTCTGTTACTAACATGTTTACTTCCATT  360
M. diversifolia GTAGCTTACCCTTTAGACCTTTTTGAAGAAGGTTCTGTTACTAACATGTTTACTTCCATT  360
M. hirsuta      GTAGCTTACCCTTTAGACCTTTTTGAAGAAGGTTCTGTTACTAACATGTTTACTTCCATT  360
M. rotundifolia GTAGCTTACCCTTTAGACCTTTTTGAAGAAGGTTCTGTTACTAACATGTTTACTTCCATT  360
                ************************************************************

M. speciosa     GTAGGTAATGTATTTGGGTTCAAAGCCCTGCGTGCTCTACGTCTGGAAGATTTGCGAATT  420
M. diversifolia GTAGGTAATGTATTTGGGTTCAAAGCCCTGCGTGCTCTACGTCTGGAAGATTTGCGAATT  420
M. hirsuta      GTAGGTAATGTATTTGGGTTCAAAGCCCTGCGTGCTCTACGTCTGGAAGATTTGCGAATT  420
M. rotundifolia GTAGGTAATGTATTTGGGTTCAAAGCCCTGCGTGCTCTACGTCTGGAAGATTTGCGAATT  420
                ************************************************************

M. speciosa     CCCGTTGCTTATACTAAAACCTTCCAAGGCCCGCCTCATGGCATCCAAGTTGAGAGAGAT  480
M. diversifolia CCCGTTGCTTATACTAAAACCTTCCAAGGCCCGCCTCATGGCATCCAAGTTGAGAGAGAT  480
M. hirsuta      CCCGTTGCTTATACTAAAACCTTCCAAGGCCCGCCTCATGGCATCCAAGTTGAGAGAGAT  480
M. rotundifolia CCCGTTGCTTATACTAAAACCTTCCAAGGCCCGCCTCATGGCATCCAAGTTGAGAGAGAT  480
                ************************************************************

M. speciosa     AAATTGAACAAGTATGGTCGTCCCCTGTTGGGATGTACTATTAAACCTAAATTAGGTTTA  540
M. diversifolia AAATTGAACAAGTATGGTCGTCCCCTGTTGGGATGTACTATTAAACCTAAATTAGGTTTA  540
M. hirsuta      AAATTGAACAAGTATGGTCGTCCCCTGTTGGGATGTACTATTAAACCTAAATTAGGTTTA  540
M. rotundifolia AAATTGAACAAGTATGGTCGTCCCCTGTTGGGATGTACTATTAAACCTAAATTAGGTTTA  540
                ************************************************************

M. speciosa     TCTGCTAAAAACTACGGTAGAGCAGTTTATGAATGTCTTCGTGGTGGGCTTGATTTTACC  600
M. diversifolia TCTGCTAAAAACTACGGTAGAGCAGTTTATGAATGTCTTCGTGGTGGGCTTGATTTTACC  600
M. hirsuta      TCTGCTAAAAACTACGGTAGAGCAGTTTATGAATGTCTTCGTGGTGGGCTTGATTTTACC  600
M. rotundifolia TCTGCTAAAAACTACGGTAGAGCAGTTTATGAATGTCTTCGTGGTGGGCTTGATTTTACC  600
                ************************************************************

M. speciosa     AAAGATGATGAAAACGTGAACTCCCAACCATTTATGCGTTGGAGAGATCGTTTCTTATTT  660
M. diversifolia AAAGATGATGAAAACGTGAACTCCCAACCATTTATGCGTTGGAGAGATCGTTTCTTATTT  660
M. hirsuta      AAAGATGATGAAAACGTGAACTCCCAACCATTTATGCGTTGGAGAGATCGTTTCTTATTT  660
M. rotundifolia AAAGATGATGAAAACGTGAACTCCCAACCATTTATGCGTTGGAGAGATCGTTTCTTATTT  660
                ************************************************************

M. speciosa     TGTGCCGAAGCACTTTATAAAGCACAGGCTGAAACAGGTGAAATCAAGGGGCATTACTTG  720
M. diversifolia TGTGCCGAAGCACTTTATAAAGCACAGGCTGAAACAGGTGAAATCAAGGGGCATTACTTG  720
M. hirsuta      TGTGCCGAAGCACTTTATAAAGCACAGGCTGAAACAGGTGAAATCAAGGGGCATTACTTG  720
M. rotundifolia TGTGCCGAAGCACTTTATAAAGCACAGGCTGAAACAGGTGAAATCAAGGGGCATTACTTG  720
                ************************************************************

M. speciosa     AATGCTACTGCAGGTACATGCGAAGAAATGATCAAAAGAGCTGTATTTGCTAGAGAATTG  780
M. diversifolia AATGCTACTGCAGGTACATGCGAAGAAATGATCAAAAGAGCTGTATTTGCTAGAGAATTG  780
M. hirsuta      AATGCTACTGCAGGTACATGCGAAGAAATGATCAAAAGAGCTGTATTTGCTAGAGAATTG  780
M. rotundifolia AATGCTACTGCAGGTACATGCGAAGAAATGATCAAAAGAGCTGTATTTGCTAGAGAATTG  780
                ************************************************************

M. speciosa     GGAGTTCCTATCGTAATGCATGATTACTTAACGGGGGGATTCACTGCAAATACTAGCTTG  840
M. diversifolia GGAGTTCCTATCGTAATGCATGATTACTTAACGGGGGGATTCACTGCAAATACTAGCTTG  840
M. hirsuta      GGAGTTCCTATCGTAATGCATGATTACTTAACGGGGGGATTCACTGCAAATACTAGCTTG  840
M. rotundifolia GGAGTTCCTATCGTAATGCATGATTACTTAACGGGGGGATTCACTGCAAATACTAGCTTG  840
                ************************************************************

M. speciosa     GCTCATTATTGCCGAGATAATGGTCTACTTCTTCACATCCACCGCGCAATGCATGCGGTT  900
M. diversifolia GCTCATTATTGCCGAGATAATGGTCTACTTCTTCACATCCACCGCGCAATGCATGCGGTT  900
M. hirsuta      GCTCATTATTGCCGAGATAATGGTCTACTTCTTCACATCCACCGCGCAATGCATGCGGTT  900
```

```
M. rotundifolia GCTCATTATTGCCGAGATAATGGTCTACTTCTTCACATCCACCGCGCAATGCATGCGGTT  900
                ************************************************************

M. speciosa     ATTGATAGGCAGAAGAATCATGGTATGCACTTTCGCGTACTAGCTAAAGCCTTACGTCTG  960
M. diversifolia ATTGATAGGCAGAAGAATCATGGTATGCACTTTCGCGTACTAGCTAAAGCCTTACGTCTG  960
M. hirsuta      ATTGATAGGCAGAAGAATCATGGTATGCACTTTCGCGTACTAGCTAAAGCCTTACGTCTG  960
M. rotundifolia ATTGATAGGCAGAAGAATCATGGTATGCACTTTCGCGTACTAGCTAAAGCCTTACGTCTG  960
                ************************************************************

M. speciosa     TCTGGTGGAGATCATATTCACGCTGGTACCGTAGTAGGGAAACTTGAAGGGGAAAGAGAC  1020
M. diversifolia TCTGGTGGAGATCATATTCACGCTGGTACCGTAGTAGGGAAACTTGAAGGGGAAAGAGAC  1020
M. hirsuta      TCTGGTGGAGATCATATTCACGCTGGTACCGTAGTAGGGAAACTTGAAGGGGAAAGAGAC  1020
M. rotundifolia TCTGGTGGAGATCATATTCACGCTGGTACCGTAGTAGGGAAACTTGAAGGGGAAAGAGAC  1020
                ************************************************************

M. speciosa     ATCACTTTGGGCTTTGTTGATTTACTGCGTGATGATTTTATTGAAAAAGATCGAAGTCGC  1080
M. diversifolia ATCACTTTGGGCTTTGTTGATTTACTGCGTGATGATTTTATTGAAAAAGATCGAAGTCGC  1080
M. hirsuta      ATCACTTTGGGCTTTGTTGATTTACTGCGTGATGATTTTATTGAAAAAGATCGAAGTCGC  1080
M. rotundifolia ATCACTTTGGGCTTTGTTGATTTACTGCGTGATGATTTTATTGAAAAAGATCGAAGTCGC  1080
                ************************************************************

M. speciosa     GGTATTTATTTCACCCAAGATTGGGTCTCTCTACCAGGTGTTCTGCCCGTGGCTTCAGGA  1140
M. diversifolia GGTATTTATTTCACCCAAGATTGGGTCTCTCTACCAGGTGTTCTGCCCGTGGCTTCAGGA  1140
M. hirsuta      GGTATTTATTTCACCCAAGATTGGGTCTCTCTACCAGGTGTTCTGCCCGTGGCTTCAGGA  1140
M. rotundifolia GGTATTTATTTCACCCAAGATTGGGTCTCTCTACCAGGTGTTCTGCCCGTGGCTTCAGGA  1140
                ************************************************************

M. speciosa     GGTATTCACGTTTGGCATATGCCTGCTTTGACCGAGATCTTTGGGGACGATTCTGTACTA  1200
M. diversifolia GGTATTCACGTTTGGCATATGCCTGCTTTGACCGAGATCTTTGGGGACGATTCTGTACTA  1200
M. hirsuta      GGTATTCACGTTTGGCATATGCCTGCTTTGACCGAGATCTTTGGGGACGATTCTGTACTA  1200
M. rotundifolia GGTATTCACGTTTGGCATATGCCTGCTTTGACCGAGATCTTTGGGGACGATTCTGTACTA  1200
                ************************************************************

M. speciosa     CAGTTCGGTGGAGGAACTTTAGGACACCCTTGGGGTAATGCGCCAGGTGCCGTAGCGAAT  1260
M. diversifolia CAGTTCGGTGGAGGAACTTTAGGACACCCTTGGGGTAATGCGCCAGGTGCCGTAGCGAAT  1260
M. hirsuta      CAGTTCGGTGGAGGAACTTTAGGACACCCTTGGGGTAATGCGCCAGGTGCCGTAGCGAAT  1260
M. rotundifolia CAGTTCGGTGGAGGAACTTTAGGACACCCTTGGGGTAATGCGCCAGGTGCCGTAGCGAAT  1260
                ************************************************************

M. speciosa     CGAGTAGCTCTAGAAGCATGTGTAAAAGCTCGTAATGAGGGGCGCGATCTTGCTGCTGAG  1320
M. diversifolia CGAGTAGCTCTAGAAGCATGTGTAAAAGCTCGTAATGAGGGGCGCGATCTTGCTTCTGAG  1320
M. hirsuta      CGAGTAGCTCTAGAAGCATGTGTAAAAGCTCGTAATGAGGGGCGCGATCTTGCTTCTGAG  1320
M. rotundifolia CGAGTAGCTCTAGAAGCATGTGTAAAAGCTCGTAATGAGGGGCGCGATCTTGCTTCTGAG  1320
                ******************************************************^*****

M. speciosa     GGTAATGAAATTATCCGTGAGGCTAGTAAATGGAGTCCTGAATTAGCTGCTGCTTGTGAG  1380
M. diversifolia GGTAATGAAATTATCCGTGAGGCTAGTAAATGGAGTCCTGAATTAGCTGCTGCTTGTGAG  1380
M. hirsuta      GGTAATGAAATTATCCGTGAGGCTAGTAAATGGAGTCCTGAATTAGCTGCTGCTTGTGAG  1380
M. rotundifolia GGTAATGAAATTATCCGTGAGGCTAGTAAATGGAGTCCTGAATTAGCTGCTGCTTGTGAG  1380
                ************************************************************

M. speciosa     GTATGGAAGGAGATCAGGTTTAATTTTAAAGCAGTGGATACTTTGGATCCGTCGTAA  1437
M. diversifolia GTATGGAAGGAGATCAGGTTTAATTTTAAAGCAGTGGATACTTTGGATCCGTCGTAA  1437
M. hirsuta      GTATGGAAGGAGATCAGGTTTAATTTTAAAGCAGTGGATACTTTGGATCCGTCGTAA  1437
M. rotundifolia GTATGGAAGGAGATCAGGTTTAATTTTAAAGCAGTGGATACTTTGGATCCGTCGTAA  1437
                *********************************************************
```

MatK

```
M. speciosa     ATGGAGGAAATCCAAAGATATTTACAGCGTGATAGATCTCAACAACACGGCTTTCTATAT  60
M. diversifolia ATGGAGGAAATCCAAAGATATTTACAGCGTGATAGATCTCAACAACACGGCTTTCTATAT  60
M. hirsuta      ATGGAGGAAATCCAAAGATATTTACAGCGTGATAGATCTCAACAACACGGCTTTCTATAT  60
M. rotundifolia ATGGAGGAAATCCAAAGATATTTACAGCGTGATAGATCTCAACAACACGGCTTTCTATAT  60
                ************************************************************

M. speciosa     CCACTTATCTTTCAGGAGTATATTTATGCACTTGCTCATGATCATAGTTTAAACCGATCT  120
M. diversifolia CCACTTATCTTTCAGGAGTATATTTATGCACTTGCTCATGATCATAGTTTAAACCGATCT  120
M. hirsuta      CCACTTATCTTTCAGGAGTATATTTATGCACTTGCTCATGATCATAGTTTAAACCGATCT  120
M. rotundifolia CCACTTATCTTTCAGGAGTATATTTATGCACTTGCTCATGATCATAGTTTAAACCGATCT  120
                ************************************************************

M. speciosa     ATTTTGTTGGAAAATCCGGGTTATGACAATCAATTCAGTTTCCTAATTGTGAAACGTTTA  180
M. diversifolia ATTTTGTTGGAAAATCCGGGTTATGACAATCAATTCAGTTTCCTAATTGTGAAACGTTTA  180
M. hirsuta      ATTTTGTTGGAAAATCCGGGTTATGACAATCAATTCAGTTTCCTAATTGTGAAACGTTTA  180
M. rotundifolia ATTTTGTTGGAAAATCCGGGTTATGACAATCAATTCAGTTTCCTAATTGTGAAACGTTTA  180
                ************************************************************
```

```
M. speciosa      ATTACTCGAATGTATCAACAAAATCATTTTATTATTTTTGCTAATGATTCTAATCAAAAT  240
M. diversifolia  ATTACTCGAATGTATCAACAAAATCATTTTATTATTTTTGCTAATGATTCTAATCAAAAT  240
M. hirsuta       ATTACTCGAATGTATCAACAAAATCATTTTATTATTTTTGCTAATGATTCTAATCAAAAT  240
M. rotundifolia  ATTACTCGAATGTATCAACAAAATCATTTTATTATTTTTGCTAATGATTCTAATCAAAAT  240
                 ************************************************************

M. speciosa      CCTTTTTTTGGTCGCAACAAGAATTTATATTCTGAAATGATATCCGAAGGATTCGCATTT  300
M. diversifolia  CCTTTTTTTGGTCGCAACAAGAATTTATATTCTGAAATGATATCCGAAGGATTCGCATTT  300
M. hirsuta       CCTTTTTTTGGTCGCAACAAGAATTTATATTCTGAAATGATATCCGAAGGATTCGCATTT  300
M. rotundifolia  CCTTTTTTTGGTCGCAACAAGAATTTATATTCTGAAATGATATCCGAAGGATTCGCATTT  300
                 ************************************************************

M. speciosa      ATTGTGGAAATTCCATTTTATATACGATTAATATCTTCTCAAGCGGGGAAAGAAATATTA  360
M. diversifolia  ATTGTGGAAATTCCATTTTATATACGATTAATATCTTCTCAAGCGGGGAAAGAAATATTA  360
M. hirsuta       ATTGTGGAAATTCCATTTTATATACGATTAATATCTTCTCAAGCGGGGAAAGAAATATTA  360
M. rotundifolia  ATTGTGGAAATTCCATTTTATATACGATTAATATCTTCTCAAGCGGGGAAAGAAATATTA  360
                 ************************************************************

M. speciosa      AAATCCTATAATTTACGATCAATTCATTCACTATTTCCTTTCTTAGAGAGCCATTTTTCA  420
M. diversifolia  AAATCCTATAATTTACGATCAATTCATTCACTATTTCCTTTCTTAGAGAGCCATTTTTCA  420
M. hirsuta       AAATCCTATAATTTACGATCAATTCATTCACTATTTCCTTTCTTAGAGAGCCATTTTTCA  420
M. rotundifolia  AAATCCTATAATTTACGATCAATTCATTCACTATTTCCTTTCTTAGAGAGCCATTTTTCA  420
                 ************************************************************

M. speciosa      CATTTAAATTCTGTGTTAGATATACTAATACCCCACCCCGTCCATCTAGAAATCTTGGTT  480
M. diversifolia  CATTTAAATTCTGTGTTAGATATACTAATACCCCACCCCGTCCATCTAGAAATCTTGGTT  480
M. hirsuta       CATTTAAATTCTGTGTTAGATATACTAATACCCCACCCCGTCCATCTAGAAATCTTGGTT  480
M. rotundifolia  CATTTAAATTCTGTGTTAGATATACTAATACCCCACCCCGTCCATCTAGAAATCTTGGTT  480
                 ************************************************************

M. speciosa      CAAAACCTTCGTTATTGGGTAAAAGACGCCCCTTCCTTGCATTTATTACGATTCTTTTTC  540
M. diversifolia  CAAAACCTTCGTTATTGGGTAAAAGACGCCCCTTCCTTGCATTTATTACGATTCTTTTTC  540
M. hirsuta       CAAAACCTTCGTTATTGGGTAAAAGACGCCCCTTCCTTGCATTTATTACGATTCTTTTTC  540
M. rotundifolia  CAAAACCTTCGTTATTGGGTAAAAGACGCCCCTTCCTTGCATTTATTACGATTCTTTTTC  540
                 ************************************************************

M. speciosa      CACGAGTATTGGAATTGGAATACTCTTATTACTAGAAAGAATCCAGTTTTTCTTTTTCA  600
M. diversifolia  CACGAGTATTGGAATTGGAATACTCTTATTACTAGAAAGAATCCAGTTTTTATTTTTCA  600
M. hirsuta       CACGAGTATTGGAATTGGAATACTCTTATTACTAGAAAGAATCCAGTTTTTATTTTTCA  600
M. rotundifolia  CACGAGTATTGGAATTGGAATACTCTTATTACTAGAAAGAATCCAGTTTTTATTTTTCA  600
                 ***************************************************^********

M. speciosa      CCAAAAAGAAATAAAAGATTATTATTCTTTTTATATAATTCTCATGTATGTGAATACGAA  660
M. diversifolia  CCAAAAAGAAATAAAAGATTATTATTCTTTTTATATAATTCTCATGTATGTGAATACGAA  660
M. hirsuta       CCAAAAAGAAATAAAAGATTATTATTCTTTTTATATAATTCTCATGTATGTGAATACGAA  660
M. rotundifolia  CCAAAAAGAAATAAAAGATTATTATTCTTTTTATATAATTCTCATGTATGTGAATACGAA  660
                 ************************************************************

M. speciosa      TCCATTTTCGTCTTTCTCCGTAACCAATCTTCTCATTTGCGATCAACATCTTTTGGAGTC  720
M. diversifolia  TCCATTTTCGTCTTTCTCCGTAATCAATCTTCTCATTTGCGATCAACATCTTTTGGAGTC  720
M. hirsuta       TCCATTTTCGTCTTTCTCCGTAATCAATCTTCTCATTTGCGATCAACATCTTTTGGAGTC  720
M. rotundifolia  TCCATTTTCGTCTTTCTCCGTAATCAATCTTCTCATTTGCGATCAACATCTTTTGGAGTC  720
                 ***********************^************************************

M. speciosa      TTTCTTGAACGAATATATTTTTATGGAAAAATAGAACGTCTTGTAGAAGTTTTTGCTAAG  780
M. diversifolia  TTTCTTGAACGAATATATTTTTATGGAAAAATAGAACGTCTTGTAGAAGTTTTTGCTAAG  780
M. hirsuta       TTTCTTGAACGAATATATTTTTATGGAAAAATAGAACGTCTTGTAGAAGTTTTTGCTAAG  780
M. rotundifolia  TTTCTTGAACGAATATATTTTTATGGAAAAATAGAACGTCTTGTAGAAGTTTTTGCTAAG  780
                 ************************************************************

M. speciosa      GATTTTCAGGCTAGTTTATGGTTGTTCAAAGATCCTTTCATGCATTATGTTAGGTATCAA  840
M. diversifolia  GATTTTCAGGCTAGTTTATGGTTGTTCAAAGATCCTTTCATGCATTATGTTAGGTATCAA  840
M. hirsuta       GATTTTCAGGCTAGTTTATGGTTGTTCAAAGATCCTTTCATGCATTATGTTAGGTATCAA  840
M. rotundifolia  GATTTTCAGGCTAGTTTATGGTTGTTCAAAGATCCTTTCATGCATTATGTTAGGTATCAA  840
                 ************************************************************

M. speciosa      GGAAAATCCATTTTGGCTTCAAAAGGGACGCCCCTTTTGATGAATAAATGGAAATATTAC  900
M. diversifolia  GGAAAATCCATTTTGGCTTCAAAAGGGACGCCCCTTTTGATGAATAAATGGAAATATTAC  900
M. hirsuta       GGAAAATCCATTTTGGCTTCAAAAGGGACGCCCCTTTTGATGAATAAATGGAAATATTAC  900
M. rotundifolia  GGAAAATCCATTTTGGCTTCAAAAGGGACGCCCCTTTTGATGAATAAATGGAAATATTAC  900
                 ************************************************************

M. speciosa      CTTGTCAATTTTTGGCAGTCTTATTTGAATCTGTGGTTTCATTCCAGAAGGGTCTATATA  960
M. diversifolia  CTTGTCAATTTTTGGCAGTGTTATTTGAATCTGTGGTTTCATTCCAGAAGGGTCTATATA  960
M. hirsuta       CTTGTCAATTTTTGGCAGTGTTATTTGAATCTGTGGTTTCATTCCAGAAGGGTCTATATA  960
M. rotundifolia  CTTGTCAATTTTTGGCAGTGTTATTTGAATCTGTGGTTTCATTCCAGAAGGGTCTATATA  960
```

```
                   *******************^****************************************

M. speciosa        AACCAATTATCCAATCATTCCCTTAACTTTCTAGGTTATCTTTCAAGTGTGCGACTAAAC  1020
M. diversifolia    AACCAATTATCCAATCATTCCCTTAACTTTCTAGGTTATCTTTCAAGTGTGCGACTAAAC  1020
M. hirsuta         AACCAATTATCCAATCATTCCCTTAACTTTCTAGGTTATCTTTCAAGTGTGCGACTAAAC  1020
M. rotundifolia    AACCAATTATCCAATCATTCCCTTAACTTTCTAGGTTATCTTTCAAGTGTGCGACTAAAC  1020
                   ************************************************************

M. speciosa        CCTTCAATGGTACGGAGTCAAATGCTAGAAAATTCATTTTTAATCAATAATGCTATTAAG  1080
M. diversifolia    CCTTCAATGGTACGGAGTCAAATGCTAGAAAATTCATTTTTAATCAATAATGCTATTAAG  1080
M. hirsuta         CCTTCAATGGTACGGAGTCAAATGCTAGAAAATTCATTTTTAATCAATAATGCTATTAAG  1080
M. rotundifolia    CCTTCAATGGTACGGAGTCAAATGCTAGAAAATTCATTTTTAATCAATAATGCTATTAAG  1080
                   ************************************************************

M. speciosa        AAATTTGATACCCTTGTTCCAATTATTCCTCTTATTAGATCATTGGCTAAAGCAAAATTT  1140
M. diversifolia    AAATTTGATACCCTTGTTCCAATTATTCCTCTTATTAGATCATTGGCTAAAGCAAAATTT  1140
M. hirsuta         AAATTTGATACCCTTGTTCCAATTATTCCTCTTATTAGATCATTGGCTAAAGCAAAATTT  1140
M. rotundifolia    AAATTTGATACCCTTGTTCCAATTATTCCTCTTATTAGATCATTGGCTAAAGCAAAATTT  1140
                   ************************************************************

M. speciosa        TGTAACCTATTAGGACATCCCGTTAGTAAGCCGGTTTGGACTGATTTATCGGATTCTGAT  1200
M. diversifolia    TGTAACCTATTAGGACATCCCGTTAGTAAGCCGGTTTGGACTGATTTATCGGATTCTGAT  1200
M. hirsuta         TGTAACCTATTAGGACATCCCGTTAGTAAGCCGGTTTGGACTGATTTATCGGATTCTGAT  1200
M. rotundifolia    TGTAACCTATTAGGACATCCCGTTAGTAAGCCGGTTTGGACTGATTTATCGGATTCTGAT  1200
                   ************************************************************

M. speciosa        ATTATTGACCGATTTGGACATATATGCAGAAACCTTTCTCATTATCATAGCGGATCTTCC  1260
M. diversifolia    ATTATTGACCGATTTGGACATATATGCAGAAACCTTTCTCATTATCATAGCGGATCTTCC  1260
M. hirsuta         ATTATTGACCGATTTGGACATATATGCAGAAACCTTTCTCATTATCATAGCGGATCTTCC  1260
M. rotundifolia    ATTATTGACCGATTTGGACATATATGCAGAAACCTTTCTCATTATCATAGCGGATCTTCC  1260
                   ************************************************************

M. speciosa        AAAAAAAAGAGTTTGTATCGAATAAAGTATATACTTCGACTTTCTTGTGCTAAAACTTTG  1320
M. diversifolia    AAAAAAAAGAGTTTGTATCGAATAAAGTATATACTTCGACTTTCTTGTGCTAAAACTTTG  1320
M. hirsuta         AAAAAAAAGAGTTTGTATCGAATAAAGTATATACTTCGACTTTCTTGTGCTAAAACTTTG  1320
M. rotundifolia    AAAAAAAAGAGTTTGTATCGAATAAAGTATATACTTCGACTTTCTTGTGCTAAAACTTTG  1320
                   ************************************************************

M. speciosa        GCTCGGAAACACAAAAGTACTGTACGTGCTTTTTTGAAAAGATTAGGCTCGGAATTTTTG  1380
M. diversifolia    GCTCGGAAACACAAAAGTACTGTACGTGCTTTTTTGAAAAGATTAGGCTCGGAATTTTTG  1380
M. hirsuta         GCTCGGAAACACAAAAGTACTGTACGTGCTTTTTTGAAAAGATTAGGCTCGGAATTTTTG  1380
M. rotundifolia    GCTCGGAAACACAAAAGTACTGTACGTGCTTTTTTGAAAAGATTAGGCTCGGAATTTTTG  1380
                   ************************************************************

M. speciosa        GAAGAATTCCTCACGTCGGAAGACGAAGTCTTTTATTTGAATTTGACCTTCCCAAGAGCT  1440
M. diversifolia    GAAGAATTCCTCACGTCGGAAGACGAAGTCCTTTATT------TGACCTTCCCAAGAGCT  1434
M. hirsuta         GAAGAATTCCTCACGTCGGAAGACGAAGTCCTTTATT------TGACCTTCCCAAGAGCT  1434
M. rotundifolia    GAAGAATTCCTCACGTCGGAAGACGAAGTCCTTTATT------TGACCTTCCCAAGAGCT  1434
                   ******************************^******^^^^^^*****************

M. speciosa        TCTTCTACTTTTCGGGGGGAATATAGAAGTCGGATTTGGTATTTGGATATTATTTATATC  1500
M. diversifolia    TCTTCTACTTTTCGGGGGGAATATAGAAGTCGGATTTGGTATTTGGATATTATTTATATC  1494
M. hirsuta         TCTTCTACTTTTCGGGGGGAATATAGAAGTCGGATTTGGTATTTGGATATTATTTATATC  1494
M. rotundifolia    TCTTCTACTTTTCGGGGGGAATATAGAAGTCGGATTTGGTATTTGGATATTATTTATATC  1494
                   ************************************************************

M. speciosa        AATGATCTGACCAATTATCAATGA  1524
M. diversifolia    AATGATCTGACCAATTATCAATGA  1518
M. hirsuta         AATGATCTGACCAATTATCAATGA  1518
M. rotundifolia    AATGATCTGACCAATTATCAATGA  1518
                   ************************
```
