# Supplementary material for: Differentiation of Mitragyna speciosa, a narcotic plant, from allied Mitragyna species using DNA barcoding-high-resolution melting (Bar-HRM) analysis
Source: Sci Rep. 2021 Mar 24;11:6738. doi: 10.1038/s41598-021-86228-9 (PMC7990970; doi:10.1038/s41598-021-86228-9)
Supplement: Supplementary file 5 — Supplementary Information 5. [file 41598_2021_86228_MOESM5_ESM.pdf]

Tittle	**Differentiation of *Mitragyna speciosa*, a narcotic plant, from allied *Mitragyna* species using DNA barcoding-high-resolution melting (Bar-HRM) analysis**
Authors	Chayapol Tungphatthong[1,3], Santhosh Kumar J. Urumarudappa1[,3], Supita Awachai[1], Thongchai Sooksawate[2] and Suchada Sukrong[1]*
Affiliation	[1]Research Unit of DNA Barcoding of Thai Medicinal Plants, Department of Pharmacognosy and Pharmaceutical Botany, Faculty of Pharmaceutical Sciences, Chulalongkorn University, Bangkok 10330, Thailand
	[2]Department of Pharmacology and Physiology, Faculty of Pharmaceutical Sciences, Chulalongkorn University, Bangkok 10330, Thailand
	[3]These authors contributed equally: Chayapol Tungphatthong and Santhosh Kumar J. Urumarudappa
*Corresponding author	Professor Suchada Sukrong, Ph.D.
	Research Unit of DNA Barcoding of Thai Medicinal Plants,
	Department of Pharmacognosy and Pharmaceutical Botany,
	Faculty of Pharmaceutical Sciences, Chulalongkorn University,
	Bangkok 10330, Thailand
	Phone: +6681-819-6742, Fax: +6622-558-227
	Email: suchada.su@chula.ac.th

Table S2: List of *Mitragyna* species occurring in Thailand along with their voucher number, collection locations, and GenBank accession number (GenBank accession number adopted from our previous study Sukrong et al., 2007 and Jaipaew et al., 2018).

**Botanical name**	**Place of collection (Province)**	**Voucher no.**	**GenBank Accession**			
			**ITS**	***mat*K**	***rbc*L**	***trn*H-*psb*A**
Mitragyna speciosa (Roxb.) Korth.	Bangkok	TTP-051001	AB249645	LC334409	LC334413	LC334417
	Bangkok	NRS-090294-597				
	Bangkok	NRS-090294-598				
	Bangkok	MUS-5512-2				
	Pathum Thani	MUS-5601-1				
	Phatthalung	MUS-5601-2				
	Satun	MUS-5601-3				
	Bangkok	NSR-090294-602				

	Bangkok	NSR-090294-603				
	Bangkok	MUS-5512-3				
	Bangkok	MUS-5601-4				
	Pathum Thani	MUS-5602-1				
	Pathum Thani	MUS-5602-2				
	Chachoengsao	MUS-5603-1				
Mitragyna diversifolia (Wall. ex G.Don) Havil.	Bangkok	NSR-090294-596	AB249646	LC334410	LC334414	LC334418
	Bangkok	MUS-5602-2				
	Suphan Buri	Suchada-041101				
	Khon Kaen	MUS-5604-1				
	Khon Kaen	MUS-5604-2				
	Nakhon Pathom	TTP-050901				
	Nakhon Pathom	MUS-5603-2				
	Nakhon Pathom	MUS-5603-3				
Mitragyna hirsuta Havil.	Bangkok	TTP-050902	AB249647	LC334412	LC334416	LC334420
	Sukhothai	TTP-051103				
	Sukhothai	MUS-5603-4				
	Kamphaeng Phet	TTP-051104				
	Kamphaeng Phet	MUS-5603-5				
	Nakhon Ratchasima	MUS-5604-3				
Mitragyna rotundifolia (Roxb.) Kuntze	Uttaradit	TTP-051105	AB249648	LC334411	LC334415	LC334419
	Bangkok	MUS-5601-5				
	Nakhon Ratchasima	MUS-5604-4				
	Nakhon Ratchasima	MUS-5604-5				
