# Supplementary material for: Differentiation of Mitragyna speciosa, a narcotic plant, from allied Mitragyna species using DNA barcoding-high-resolution melting (Bar-HRM) analysis
Source: Sci Rep. 2021 Mar 24;11:6738. doi: 10.1038/s41598-021-86228-9 (PMC7990970; doi:10.1038/s41598-021-86228-9)
Supplement: Supplementary file 6 — Supplementary Information 6. [file 41598_2021_86228_MOESM6_ESM.pdf]

Tittle	**Differentiation of *Mitragyna speciosa*, a narcotic plant, from allied *Mitragyna* species using DNA barcoding-high-resolution melting (Bar-HRM) analysis**
Authors	Chayapol Tungphatthong[1,3], Santhosh Kumar J. Urumarudappa1[,3], Supita Awachai[1], Thongchai Sooksawate[2] and Suchada Sukrong[1]*
Affiliation	[1]Research Unit of DNA Barcoding of Thai Medicinal Plants, Department of Pharmacognosy and Pharmaceutical Botany, Faculty of Pharmaceutical Sciences, Chulalongkorn University, Bangkok 10330, Thailand
	[2]Department of Pharmacology and Physiology, Faculty of Pharmaceutical Sciences, Chulalongkorn University, Bangkok 10330, Thailand
	[3]These authors contributed equally: Chayapol Tungphatthong and Santhosh Kumar J. Urumarudappa
*Corresponding author	Professor Suchada Sukrong, Ph.D.
	Research Unit of DNA Barcoding of Thai Medicinal Plants,
	Department of Pharmacognosy and Pharmaceutical Botany,
	Faculty of Pharmaceutical Sciences, Chulalongkorn University,
	Bangkok 10330, Thailand
	Phone: +6681-819-6742, Fax: +6622-558-227
	Email: suchada.su@chula.ac.th

Table S3: Collection of suspicious Kratom samples used in this study

**Sl. No.**	**Sample form**	**Place of collection (Province)**	**Sample Code.**
1	Fresh leaves	Nakhon Si Thammarat	K-01
2	Dried leaves	Nonthaburi	K-02
3	Dried leaves	Nonthaburi	K-03
4	Powders	Patthalung	K-04
5	Juices	Satun	K-05
6	Cocktails	Trang	K-06
