# Supplementary material for: Differentiation of Mitragyna speciosa, a narcotic plant, from allied Mitragyna species using DNA barcoding-high-resolution melting (Bar-HRM) analysis
Source: Sci Rep. 2021 Mar 24;11:6738. doi: 10.1038/s41598-021-86228-9 (PMC7990970; doi:10.1038/s41598-021-86228-9)
Supplement: Supplementary file 7 — Supplementary Information 7. [file 41598_2021_86228_MOESM7_ESM.pdf]

| **Tittle** | **Differentiation of *Mitragyna speciosa*, a narcotic plant, from allied *Mitragyna* species using DNA barcoding-high-resolution melting (Bar-HRM) analysis** |
|---|---|
| Authors | Chayapol Tungphatthong[1,3], Santhosh Kumar J. Urumarudappa1[,3], Supita Awachai[1], Thongchai Sooksawate[2] and Suchada Sukrong[1]* |
| Affiliation | [1]Research Unit of DNA Barcoding of Thai Medicinal Plants, Department of Pharmacognosy and Pharmaceutical Botany, Faculty of Pharmaceutical Sciences, Chulalongkorn University, Bangkok 10330, Thailand |
| | [2]Department of Pharmacology and Physiology, Faculty of Pharmaceutical Sciences, Chulalongkorn University, Bangkok 10330, Thailand |
| | [3]These authors contributed equally: Chayapol Tungphatthong and Santhosh Kumar J. Urumarudappa |
| *Corresponding author | Professor Suchada Sukrong, Ph.D. |
| | Research Unit of DNA Barcoding of Thai Medicinal Plants, |
| | Department of Pharmacognosy and Pharmaceutical Botany, |
| | Faculty of Pharmaceutical Sciences, Chulalongkorn University, |
| | Bangkok 10330, Thailand |
| | Phone: +6681-819-6742, Fax: +6622-558-227 |
| | Email: suchada.su@chula.ac.th |

**Table S4:** List of different DNA barcode regions and with their primer details used in this study.

| **Sl. No** | **Region with primer names** | | **Sequence (5'-3')** | **Reference** |
|---|---|---|---|---|
| 1 | *psb*A-*trn*H | trnHF | CGC GCA TGG TGG ATT CAC AAT CC | 1 |
| | | psbA-diR | GTA ATG CAT GAA CGT AAT GCT C | |
| 2 | *mat*K | trnK3914F | TGG GTT GCT AAC TCA ATG G | 2 |
| | | trnK2R | AAC TAG TCG GAT GGA GTA C | |
| | | matKMit-56F | CTT TGG TTT GAC TAT ATC GCA CTA | 3 |
| | | matKMit483F | CCC ACC CCG TCC ATC TA | |
| | | matKMit1166R | CGG CTT ACT AAC GGG ATG TC | |
| 3 | ITS | 18S-25SF | GTA GGT GAA CCT GCA GAA GGA TCA | 4 |
| | | 18S-25SR | CCA TGC TTA AAC TCA GCG GGT | |
| 4 | *rbc*L | rbcLMitF1 | TGT CAC CAC AAA CAG AAA CTA AAG CAA GT | 3 |
| | | rbcLMitR1 | CTT TTA GTA AAG ATT GGG CCG AG | |
| | | rbcLMitF2 | CGA GTA GCT CTA GAA GCA TGT GTA AAA G | 3 |
| | | rbcLMitR2 | TTT GTA ACG ATC AAG GCT GGT AAG TAA | |
| 5 | ITS2 | Mitragyna_ITS2_F | CGG CCT AAA TGC GAG TCC TC | This study |
| | | Mitragyna_ITS2_R | CGG CAC GAC AGA AAT CGA GTC | |
| 6 | *mat*K | Mitragyna_matK_F | TGTGAATACGAATCCATTTTCGTC | This study |
| | | Mitragyna_matK_R | ACTCCAAAAGATGTTGATCGCA | |
| 7 | *rbc*L | Mitragyna_rbcL_F | AAAGCTCGTAATGAGGGGCG | This study |
| | | Mitragyna_rbcL_R | ACTCCATTTACTAGCCTCACGG | |
| 8 | *psbA*-trn*H* | Mitragyna_psbA_F | AGAAGAAGAAGGAAATTATTGCTCC | This study |
| | | Mitragyna_psbA_R | CCTAGCTGCTATAGATGCTCCA | |

**Reference:**


1. Vongsak, B., Kengtong, S., Vajrodaya, S., & Sukrong, S. Sequencing analysis of the medicinal plant Stemona tuberosa and five related species existing in Thailand based on trnH-psbA chloroplast DNA. *Planta Med* 74(14), 1764-1766 (2008).
2. Johnson, L. A., & Soltis, D. E. matK DNA sequences and phylogenetic reconstruction in Saxifragaceae s. str. *Syst. Bot* 143-156 (1994).
3. Jaipaew, J., Padungchareon, T., & Sukrong, S. PCR-reverse dot blot of the nucleotide signature sequences of matK for the identification of *Mitragyna speciosa*, a narcotic species. *Plant Gene* 14, 46-54 (2018).
4. Takaiwa, F., Oono, K., & Sugiura, M. Nucleotide sequence of the 17S–25S spacer region from rice rDNA. *Plant Mol. Biol* 4(6), 355-364 (1985).
